# Hypoxia inducible factor prolyl hydroxylases in inflammatory bowel disease

**DOI:** 10.3389/fphar.2023.1045997

**Published:** 2023-05-02

**Authors:** Jie Lun, Hongwei Zhang, Jing Guo, Mengchao Yu, Jing Fang

**Affiliations:** ^1^ Department of Oncology, Cancer Institute, The Affiliated Hospital of Qingdao University, Qingdao, China; ^2^ Shandong Provincial Maternal and Child Health Care Hospital Affiliated to Qingdao University, Jinan, China; ^3^ Department of Gastroenterology, Qingdao Municipal Hospital, Qingdao, China

**Keywords:** prolyl hydroxylases, hypoxia inducible factor, inflammatory bowel disease, intestine epithelial, barrier function, inhibitor, therapeutics

## Abstract

Inflammatory bowel disease (IBD) is a chronic disease that is characterized by intestinal inflammation. Epithelial damage and loss of intestinal barrier function are believed to be the hallmark pathologies of the disease. In IBD, the resident and infiltrating immune cells consume much oxygen, rendering the inflamed intestinal mucosa hypoxic. In hypoxia, the hypoxia-inducible factor (HIF) is induced to cope with the lack of oxygen and protect intestinal barrier. Protein stability of HIF is tightly controlled by prolyl hydroxylases (PHDs). Stabilization of HIF through inhibition of PHDs is appearing as a new strategy of IBD treatment. Studies have shown that PHD-targeting is beneficial to the treatment of IBD. In this Review, we summarize the current understanding of the role of HIF and PHDs in IBD and discuss the therapeutic potential of targeting PHD-HIF pathway for IBD treatment.

## 1 Introduction

Inflammatory bowel disease (IBD) is a chronic inflammatory disorder of the gastrointestinal tract (Zhang and Li, 2014). The major types of IBD are Crohn’s disease (CD) and ulcerative colitis (UC). IBD is debilitating and associated with the development of a number of complications. It affects millions of people worldwide with higher incidence in developed countries (Loftus, 2004). The pathology of IBD is complex and may involve a combination of genetic, environmental factors and immunological abnormalities (Kaser et al., 2010; Ananthakrishnan et al., 2018). The core pathology of IBD is believed to be the disruption of epithelial barrier that separates the intestinal lumen from the mucosal immune system. The impairment of intestinal barrier leads to exposure of mucosal immune cells to microorganisms and antigens, which causes inflammation and ruins the integrity of the intestinal barrier, resulting in progressive and cyclical inflammation as well as a long-term damage to the intestine (Brown and Taylor, 2018). Currently, antibiotics, anti-inflammatory agents and surgery are employed in the treatment of IBD. The effectiveness of these treatments is variable and usually unsatisfactory. There is an unmet medical need for this disease.

Compared with other tissues, intestine is hypoxic and intestinal inflammation exacerbates the lack of oxygen (Taylor and Colgan, 2017). Mucosal hypoxia is an integral component of IBD. The intestine is highly dependent on the adaptive pathways activated by hypoxia. Studies have revealed that hypoxia-inducible factor (HIF) protects intestinal barrier and elicits anti-inflammatory responses. Protein stability of HIF is tightly regulated by prolyl hydroxylases (PHDs). Induction of expression of HIF through inhibition of PHDs has been shown beneficial to IBD treatment (Manresa and Taylor, 2017; Van Welden et al., 2017a). Herein, we review the current understanding of the impacts of HIF and PHDs on IBD and discuss the therapeutic potential of targeting PHD-HIF axis for the treatment of this disease.

## 2 IBD and hypoxia

Hypoxia is a feature of the intestinal mucosa. Under normal conditions, there is a steep oxygen gradient from the anaerobic lumen to the oxygen-rich submucosa in the gastrointestinal tract (Taylor and Colgan, 2007). The oxygen contents in the small intestinal wall and the villus tip are about 8% and 3%, respectively. The oxygen content in gut lumen is less than 2%, whereas the arteries in the submucosa have an oxygen level around 80%–100% (He et al., 1999; Fisher et al., 2013; Zeitouni et al., 2016).

Due to increased cell metabolism and decreased supply of oxygen, the inflamed regions are usually short of oxygen (Fraisl et al., 2009; Eltzschig and Carmeliet, 2011; Bartels et al., 2013; Eltzschig et al., 2014). During inflammation, the resident immune cells such as macrophages and dendritic cells are activated. These activated immune cells produce proinflammatory cytokines and chemokines, which induces differentiation of T cells and recruits inflammatory cells from blood to mucosa. The infiltrated immune cells and intestinal epithelial cells in the inflamed regions consume a large amount of oxygen (Campbell et al., 2014). In the meantime, the microthrombosis in inflamed tissues may cause decreased oxygen supply from the bloodstream (Hatoum et al., 2003). The increased oxygen consumption and decreased oxygen supply result in lack of oxygen in the inflamed mucosa. Multiple IBD models have demonstrated that the inflamed intestinal mucosa is short of oxygen and hypoxia is a common feature in the inflamed mucosa in IBD (Taylor and Colgan, 2017).

## 3 Hypoxia inducible factor (HIF) α and prolyl hydroxylase (PHD)

**FIGURE 1:**
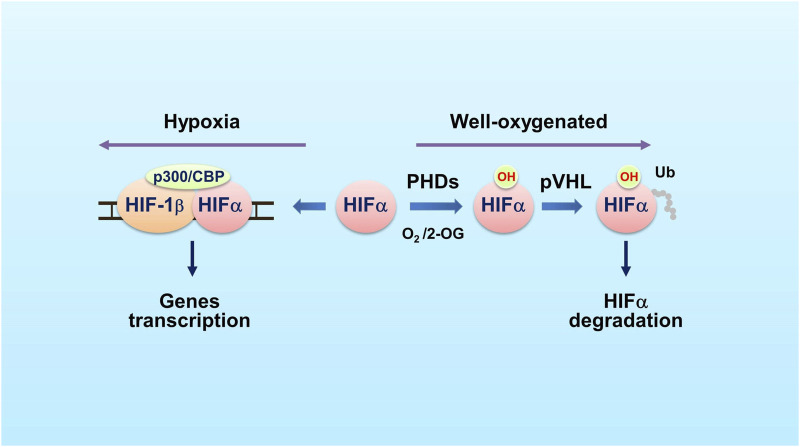
The PHD-HIF oxygen-sensing system. In well oxygenated cells (schematic on the right side), PHDs hydroxylate HIFα on specific proline residues using O_2_ and 2-OG as co-substrates. The E3 ubiquitin ligase pVHL recognizes the hydroxylated HIFα and mediates HIFα ubiquitination and proteasomal degradation. In hypoxic cells (left side), the PHDs’ prolyl hydroxylase activity is inhibited, leading to HIFα accumulation. HIFα then dimerizes with HIF-1β and recruits CBP and p300 co-factors. The complex binds to hypoxia response element (HRE) within or near target genes to activate transcription of these genes. Abbreviations used: HIF, hypoxia-inducible factor; PHD, prolyl hydroxylase; Ub, ubiquitin; pVHL, the von Hippel-Lindau protein; 2-OG, 2-oxoglutarate.

In the intestine, the adaptation of the cells to the lack of oxygen is regulated by HIF. HIF is a basic helix-loop-helix-PAS domain transcription factor that is composed of an alpha subunit (HIF-1α, -2α and -3α) and a constitutively expressed beta subunit (known as HIF-1β) (Wang et al., 1995). HIFα binds HIF-1β to form an active transcription factor. The transcription factor recruits co-factors p300 and CBP and the complex binds hypoxia responsive elements (HRE) within or near target genes to initiate transcription of the genes that are involved in cell survival, angiogenesis and metabolism (Figure 1) (Schofield and Ratcliffe, 2004; Chowdhury et al., 2008; Ortiz-Barahona et al., 2010; Biddlestone et al., 2015; Taylor and Scholz, 2022; Wicks and Semenza, 2022).

Stability of HIFα is controlled by PHDs. In the presence of oxygen, PHDs hydroxylate the highly conserved proline residues of HIFα. The hydroxylated HIFα is then recognized by the von Hippel-Lindau (pVHL) protein, an E3 ubiquitin ligase, and is ubiquitinated for degradation (Maxwell et al., 1999; Ivan et al., 2001) (Figure 1). PHDs are dioxygenases that use O_2_ and 2-oxoglutarate (2-OG) as co-substrates and Fe^2+^ and ascorbic acid as co-factors. The PHD family has three members: PHD1 (EglN2), PHD2 (EglN1) and PHD3 (EglN3). PHD1, PHD2 and PHD3 share conserved C-terminal regions that are responsible for the prolyl hydroxylase activity. However, these enzymes have great differences at N terminus (Epstein et al., 2001). Each protein has its tissue- and cell-specific expression pattern as well as particular cellular distribution (Metzen et al., 2003).

Oxygen is the most important factor that controls prolyl hydroxylation catalyzed by PHDs. In hypoxia, the PHDs’ enzymatic activities are inhibited, leading to accumulation of HIFα and subsequent expression of HIFα target genes. The affinity of PHDs to oxygen is relatively low and this makes PHDs sensing oxygen in a physiologically relevant concentration range (Kaelin and Ratcliffe, 2008). Recent studies indicate that the PHDs oxygen K_M_ values are close to 100 μmol/L (Koivunen et al., 2006; Ehrismann et al., 2007), which are higher than tissue oxygen concentrations (10–30 μmol/L). The oxygen content in cells is smaller than the apparent K_M_ for oxygen, allowing that the enzymatic activities of PHDs are strictly controlled by oxygen content over the entire physiologic range (Kaelin and Ratcliffe, 2008). This characteristic makes PHDs good oxygen sensors in tissues. The PHDs and HIFα are the core of the oxygen-sensing machinery in metazoans.

Although all PHD isoforms hydroxylate HIF-1α and HIF-2α, they have differential selectivity in relation to the hydroxylation of them (Appelhoff et al., 2004). PHD2 has a greater influence on the expression of HIF-1α than that of HIF-2α, while PHD1 and PHD3 have a greater effect on the expression of HIF-2α (Appelhoff et al., 2004). Of the PHD family members, PHD2 appears to be the primary HIFα prolyl hydroxylase and the key oxygen sensor (Berra et al., 2003; Minamishima et al., 2008; Takeda et al., 2008). The *PHD2* and *PHD3* genes have HRE in their promoter regions and their transcription can be modulated by oxygen contents (Metzen et al., 2005; Pescador et al., 2005). Under hypoxia, expression of *PHD2* and *PHD3* genes is activated and the increased PHD2 and PHD3 proteins are believed to decrease the HIF response to chronic hypoxia, which may limit HIFα protein levels under hypoxia. Although the enzymatic activities of PHD2 and PHD3 are inhibited in hypoxic conditions, their presence may cause immediately the degradation of HIFα once the oxygen levels increase, thereby forming a negative feedback regulation (Stiehl et al., 2006). Interestingly, the expression of *PHD1* gene is not regulated by such a feedback mechanism (Marxsen et al., 2004).

## 4 Role of HIFα in IBD

### 4.1 HIFα in intestinal epithelial barrier

The intestine epithelial cells play an important role in defensing against microorganisms in the intestinal tract. In intestine epithelial cells, HIF-1α and HIF-2α are demonstrated to be critical in keeping barrier function and wound healing capacity (Karhausen et al., 2004; Ramakrishnan and Shah, 2016). The intestinal barriers are dynamic in nature. They are maintained mostly by mucus layer, intercellular tight junction (TJ) and adherens junction (AJ). Many investigations have demonstrated that HIFs have a barrier-protective function in the intestine (Furuta et al., 2001; Synnestvedt et al., 2002; Eltzschig et al., 2003). Both HIF-1α and HIF-2α are found expressed in human and mice intestine epithelial cells (Giatromanolaki et al., 2003; Glover et al., 2013; Xue et al., 2013). The involvement of HIF-1α and HIF-2α in the protection of intestinal epithelial barrier is well investigated and the results indicate that they are key regulators.

**FIGURE 2:**
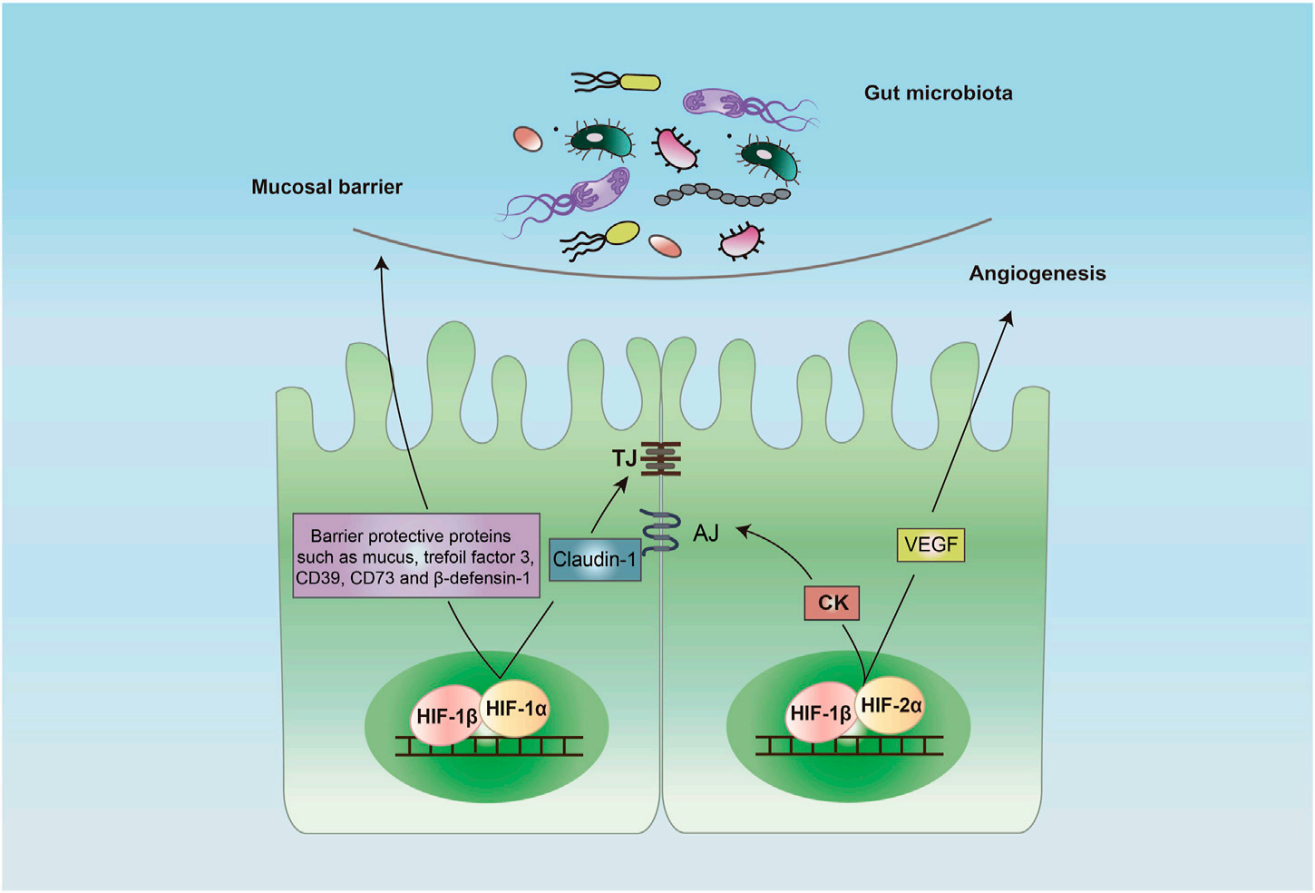
Protective role of HIF in intestinal epithelial barrier. Activation of HIF-1 in the intestine epithelial cells induces a barrier-protective pathway by increasing the expression of barrier-protective proteins such as mucus, trefoil factor 3, CD39, CD73 and β-defensin-1 and TJ protein claudin-1. Activation of HIF-2 promotes the expression of CK and VEGF that promotes AJ and angiogenesis, respectively. Abbreviations: AJ, adhesion junction; CK, creatine kinase; TJ, tight junction; VEGF, vascular endothelial growth factor.

Studies of active inflammation in mouse models of IBD have shown the intestinal epithelial cell to be a primary target for hypoxia (Karhausen et al., 2004). Strong evidence has demonstrated that HIF-1α plays a crucial role in the maintenance of intestinal barrier, and it is widely regarded as a protective factor (Figure 2), making it a potential therapeutic target for IBD (Colgan and Taylor, 2010). A major line of defense to the gut microorganisms and other pathogens is the production of mucus (Hansson, 2020). The mucus is the first barrier that gut microbes and pathogens meet. The intestine goblet cells are a kind of epithelial cells that produce and secrete mucus. Several mucins such as mucin-3 and muc5ac are the major glycoproteins in mucus and expression of these mucins are regulated by HIF-1α (Louis et al., 2006; Young et al., 2007). In addition to the mucus layer, TJ forms the core mechanism regulating the integrity of intestinal barrier. Claudin-1 is a major tight junctional protein, and its expression is directly regulated by HIF-1 (Saeedi et al., 2015). Inactivation of HIF-1 resulted in a defect in the formation of tight barriers, which was redeemed by the expression of claudin-1.

Stabilization of HIF through inhibition of PHDs promoted intestinal fibroblast-mediated collagen gel contraction, an important step in the wound-healing process (Ngo et al., 2006). It was demonstrated that pharmacological activation of HIF increased contraction of collagen gels seeded with mouse embryonic fibroblast NIH-3T3 cells (Robinson et al., 2008). Further studies using human CCD-18CO intestinal fibroblast cells were performed. When seeded in collagen gels, the cells showed a significant increase contraction when treated with PHD inhibitors, and this contraction correlated directly with increases in HIF stabilization (Keely et al., 2009). Mechanistically, HIF-1 induced expression of fibroblast integrin beta one and controlled fibroblast contraction during intestinal wound healing. HIF may also promote intestinal epithelial healing through induction of α-integrin (Goggins et al., 2021). It was shown that HIF-1α induced expression of integrins α6 and α2 to promote intestinal epithelial migration and proliferation, which played an important role in epithelial restitution. These results indicated that PHDs inhibitor stabilized HIF-1α and accelerated intestinal mucosal healing by inducing epithelial integrin expression.

In addition to direct regulation of the intestinal barrier, HIF-1α mediates several indirect mechanisms to maintain the barrier integrity. The expression of intestinal trefoil factor 3, a barrier protective protein, is regulated directly by HIF-1α (Furuta et al., 2001). Trefoil factor 3 plays an important role in repairing epithelial surfaces. Expression of CD39 and CD73, two important membrane-bound proteins, are regulated by HIF-1α. CD39 is associated with the conversion of ATP/ADP to AMP, and CD73 is involved in the degradation of AMP to adenosine (Allard et al., 2017). The CD39-and CD73-mediated degradation of ATP is critical for restoring the barrier (Synnestvedt et al., 2002). There are anti-microbial peptides in mucus layer. Beta-defensin-1, an anti-microbial peptide that is secreted by intestine epithelial cells into the mucus layer, protects against commensal overgrowth and pathogen infiltration. HIF-1α plays a critical role in the induction of production of β-defensin-1 in intestinal epithelial cells (Kelly et al., 2013). The epithelial HIF-1 may also preserve the intestinal barrier function through the induction of expression of other barrier-protective genes such as CD55 (Louis et al., 2005) and netrin-1 (Rosenberger et al., 2009), and the enhancement of extracellular adenosine signaling while inhibiting the expression of adenosine transporters and the genes related to epithelial cell death such as FADD (Hindryckx et al., 2010).

In mouse model studies, the deletion of HIF-1α in intestine epithelial cells demonstrated a major defect in the integrity of mucosal barrier. Deletion of HIF-1α in mice intestine epithelial cells showed that the absence of HIF-1α caused more severe 2,4,6-trinitrobenzene sulfonic acid (TNBS)-induced colitis, while the constitutive HIF-1α activation was protective (Karhausen et al., 2004). The mice lacking HIF-1α in intestine epithelial cells were more sensitive to bacterial toxin with a more severe colitis phenotype as compared to the control mice (Hirota et al., 2010). Together, these data suggest that epithelial HIF-1 plays a critical role in intestinal barrier protection.

HIF-2α also plays an important role in keeping homeostasis of the intestinal barrier. It regulates cell metabolism and proliferation that are required for repair of intestine epithelial injury, which is essential for highly regenerative intestinal epithelium (van der Flier and Clevers, 2009). It was reported that HIF-2α promoted the expression of creatine kinases (CKs) including CKB (brain type) and CKM (muscle type), the enzymes that are critical for rapid ATP production in intestine epithelial cells (Glover et al., 2013). The authors showed that CKs were localized to the apical intestinal epithelial cell AJ, where they were important for the AJ assembly and epithelium integrity. In a radiation-induced model of intestinal injury in mice, activation of HIF-2α was found protective by increasing crypt regeneration in vascular endothelial growth factor (VEGF)- and angiogenesis-dependent manners (Taniguchi et al., 2014). It should be noted that, in some studies, HIF-2α was found to have detrimental effects in animal models of bowel inflammation (Xue et al., 2013; Solanki et al., 2019). The role of HIF-2α in IBD remains unclear and further studies are warranted.

In hypoxia, cells shift mitochondrial respiration to glycolysis. The hypoxia-induced HIF-1α plays an important role in this metabolic switch through inducing the expression of glucose transporters and glycolytic enzymes (Seagroves et al., 2001; Kierans and Taylor, 2021), and through suppressing mitochondrial oxidative phosphorylation (Kim et al., 2006; Papandreou et al., 2006). The HIF-1α-promoted glycolysis may enhance the production and release of lactate, leading to acidification of the extracellular microenvironment (Pavlova et al., 2022), which may impact the metabolism of gut microbial communities (Taylor et al., 2022). A recent report demonstrated that the intestinal HIF-2α also positively regulated gut lactate by controlling the expression of intestinal LDHA, which shaped the gut microbiome (Wu et al., 2021). It was shown that treatment with lactate-producing *Saccharomyces cerevisiae* modulated gut microbiota and attenuated dextran sulfate sodium (DSS)-induced colitis in mice (Sun et al., 2021). Thus, activation of HIF through inhibition of PHDs may regulate intestinal barrier function through the induction of lactate and the modulation of gut microbiota.

### 4.2 HIFα in innate and adaptive immunity

**FIGURE 3:**
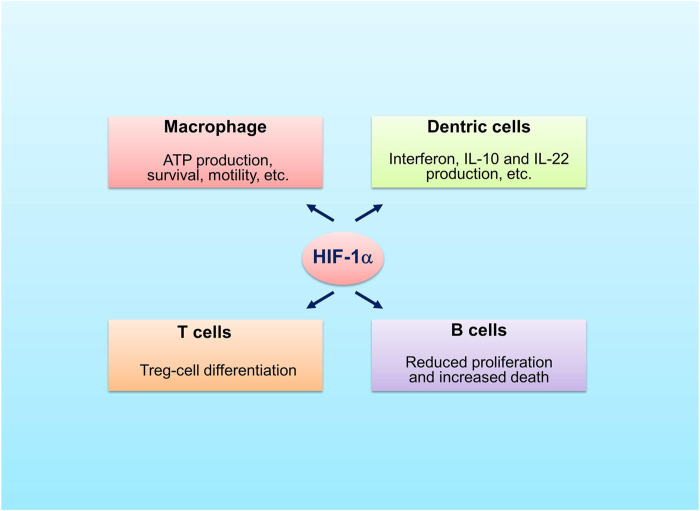
Roles of HIF-1α in immune cells. HIF-1α promotes survivability and motility of macrophage, stimulates production of interferon, IL-22 and IL-10 by dentric cells, favors differentiation of Treg cells and decreases proliferation and increases death of B cells.

Hypoxia influences immunity through induction of expression of HIFα (Dvornikova et al., 2023). HIF-1α was shown to promote neutrophil survival and enhance glycolysis (Cramer et al., 2003; Walmsley et al., 2005) (Figure 3). The macrophages rely on glycolysis to produce ATP, which is also regulated by HIF-1α (Cramer et al., 2003). The mice macrophages lacking HIF-1α cannot produce sufficient ATP, which may disserve the capability of survivability, motility, invasiveness and bacterial killing of these cells (Cramer et al., 2003). In dentric cells activation of HIF-1α promotes production of interferon, IL-22 and IL-10 and induces cell differentiation as well as cell migration (Köhler et al., 2012; Naldini et al., 2012; Wobben et al., 2013). In DSS colitis model, the mice that HIF-1α was specifically knocked out in dentric cells were more sensitive to DSS treatment as compared to the control mice (Flück et al., 2016). This was related to disrupted development of regulatory T cells (Tregs), which was caused by decreased formation of dentric cell-induced C–C chemokine receptor type 9, a marker of gut-homing T-cells, and by decreased expression of aldehyde dehydrogenase 1a2, an enzyme involved in Tregs induction. HIF-2α is also involved in the regulation of macrophages and natural killer (NK) cells. Fang et al. demonstrated that HIF-2α induced the expression of cell surface receptors and tumor-promoting cytokines in human and murine macrophages as HIF-1α did in hypoxia (Fang et al., 2009). Zhang et al. showed that HIF-2α limited NK cell cytotoxicity (Zhang et al., 2016), which indicates that HIF-2α may have an anti-inflammatory role.

HIFs are also implicated in the regulation of adaptive immune cells, including T cells and B cells. It was shown that the T cell-specific HIF-1α knockout mice had more severe gut inflammation with increased amounts of TH1 and TH17 cells when treated with DSS, implying that HIF-1α favors the differentiation of Tregs (Higashiyama et al., 2012). A recent report demonstrated that stabilization of HIF-1α enhanced the production of IL-10 and IL-22 from lamina propria CD4^+^ T-cells with reduction of inflammatory lesions in DSS-induced mice colitis (Kim et al., 2021). The Tregs without HIF-1α could not control T cell-mediated colitis (Clambey et al., 2012). Different from HIF-1α, overactivation of HIF-2α was shown to have deleterious role in the control of Tregs function (Yamamoto et al., 2019; Ajouaou et al., 2022). The findings are different from a publication by Hsu et al. (2020) in which deletion of HIF2α, but not HIF1α, was found to affect Tregs function negatively. Of note, concomitant deletion of both HIF-1α and HIF-2α restored the suppressive activity of Tregs (Hsu et al., 2020). These studies indicate that the role of HIF-2α is ambiguous. In B cells, HIF-1α regulates the expression of alkaline pH-activated two-pore domain K+ channel K2P5.1, which is required to affect B-cell proliferation, survival, or production of cytokines (Shin et al., 2014). HIF-1α acts as a transcription factor controlling the formation of IL-10 in B cells (Meng et al., 2018). Specific deletion of pVHL in B cells stabilized HIFα, leading to decreased proliferation and increased death of B cells, and impaired formation of high-affinity IgG (Cho et al., 2016).

Intestinal microenvironment acidosis also influences immune cells. Studies have shown that lactate acidosis induced by HIF-1α exerted immunomodulatory pleiotropic effects that modulate the inflammatory response (Manosalva et al., 2022). Long-term exposure to lactate results in strong anti-inflammatory effects in monocytes (Ratter et al., 2018). The anti-inflammatory effect by lactate was also observed in macrophage (Yang et al., 2020) and mast cells (Caslin et al., 2019). Lactate-driven macrophage polarization in the inflammatory microenvironment alleviates intestinal inflammation (Zhou et al., 2022). Past studies showed that lactate suppressed the innate immunity (Hoque et al., 2014) and lactate treatment protected mice against TNBS-induced colitis (Iraporda et al., 2016). Extracellular acidosis suppresses T cell-mediated immunity (Certo et al., 2021).

## 5 Role of PHDs in IBD

Studies have indicated that the three PHD family members have different roles in IBD (Watts and Walmsley, 2019; Dvornikova et al., 2023). In a DSS-induced colitis animal model, the intestinal inflammation was diminished in *Phd1*-deficient (*Phd1* ^_/_^) but unaltered in *Phd2*-deficient (*Phd2* ^+/_^) and *Phd3*-deficient (*Phd3* ^_/_^) mice, suggesting that loss of *Phd1*, but not *Phd2* or *Phd3*, is protective against DSS-induced colitis (Tambuwala et al., 2010; Kennel et al., 2022). We found that deletion of PHD2 in mice intestinal epithelial cells did not lead to spontaneous enteritis or colitis, nor did it confer upon mice higher susceptibility to DSS-induced colitis (Xie et al., 2018). While the mice with depletion of PHD3 in intestinal epithelial cells developed spontaneous colitis and were more sensitive to DSS treatment than the wild-type littermate controls, suggesting that PHD3 is protective against colitis (Chen et al., 2015). Interestingly, PHD3 was found to protect the intestinal epithelial barrier through stabilizing the TJ protein occludin (Chen et al., 2015) and the transcription factor ATOH1 (Xu et al., 2020), in a hydroxylase-independent manner. These results suggest that the PHD isoforms have different roles in IBD and they may function in different mechanisms.

Expression of PHD family members in inflamed intestinal tissues was determined. PHD1 levels were increased with disease severity in intestinal tissues from patients with IBD and in colonic tissues from mice with colitis (Tambuwala et al., 2010). Similarly, both mRNA and protein levels of PHD1 were found upregulated in inflamed biopsies from both UC and CD patients, while expression of PHD2 in colonic mucosa was not altered in IBD and expression of PHD3 was increased in inflamed biopsies from UC patients only at the mRNA level (Van Welden S, et al., 2013). These findings are consistent with the results that deletion of PHD1 is protective. A recent study demonstrated that PHD1 was downregulated in the mucosa in UC patients with active inflammatory disease, which might skew the hypoxic response toward enhanced protective HIF-1α stabilization in the inflamed mucosa of UC patients (Brown et al., 2020). In another study using the chemical-induced colitis mice model, expression of PHD1 and PHD2 was increased with the progression of the disease, while the expression of PHD3 remained unchanged (Bakshi et al., 2019). Examination of biopsies from UC patients indicated that PHD3 protein levels in inflamed mucosa were decreased with disease severity, which was consistent with the finding that PHD3 was protective against colitis in mice (Chen et al., 2015).

The possible roles of PHDs in other types of cell on IBD were also determined. Van Welden et al. (2017b) demonstrated that *Phd1* deletion in endothelial and haematopoietic cells (*Phd1* ^f/f^Tie2:cre) protected mice from DSS-induced colitis, whereas the response of *Phd2* ^*f/+*^Tie2:cre and *Phd3* ^f/f^Tie2:cre mice to DSS was similar to that of their littermate controls. While, in another study using a Crohn’s like ileitis mouse model, it was shown that haematopoietic *Phd1*-deletion did not impact experimental ileitis development (De Galan et al., 2021). It was demonstrated recently that mice lacking PHD2 expression in Tregs displayed a proinflammatory phenotype (Ajouaou et al., 2022). Deletion of PHD3 in neutrophils was found to be associated with reduced bowel inflammation in an acute mouse model of colitis (Walmsley et al., 2011). Together, these results indicate that the role of PHD isoforms may be cell-specific.

## 6 PHD inhibitors for IBD treatment

### 6.1 PHD inhibitors in experimental IBD

The protection of intestinal epithelial barrier by HIFα in IBD has initiated the study of PHDs-targeting as a strategy for IBD treatment. A few PHD inhibitors have been investigated in the treatment of gut inflammation in pre-clinical animal models (Table 1). In 2008, two studies demonstrated the protective effects of the PHD inhibitors dimethyloxalyl glycine (DMOG) (Cummins et al., 2008) and FG-4497 (Robinson et al., 2008) in experimental colitis of mice. Besides mice chemical colitis experiments, treatment with DMOG was also found beneficial in protection against bacterial toxin-, ischaemia and reperfusion-, and radiation-induced intestinal injury (Hirota et al., 2010; Hart et al., 2011; Taniguchi et al., 2014), and the protection was HIF-dependent. DMOG is an analogue of 2-OG and it blocks the entry of the co-substrate to the catalytic domain of PHDs, thus inhibiting PHDs’ enzymatic activity. FG-4497 blocks the active site of PHDs.

**TABLE 1:** PHD inhibitors in treatment of gut inflammation in animal models.

PHD inhibitors	Mechanism	IBD models	References
DMOG	2-OG analogue	DSS-induced mice colitis	Cummins et al. (2008)
		*Clostridium difficile*-induced mice gut injury	Hirota et al. (2010)
		Ischaemia–reperfusion mice gut injury	Hart et al. (2011)
		Radiation-induced mice intestinal toxicity	Taniguchi et al. (2014)
FG-4497	PHD active site blocker	TNBS-induced mice colitis	Robinson et al. (2008)
TRC160334	Unknown	DSS-induced and TNBS-induced mice colitis	Gupta et al. (2014)
Rosmarinic acid methyl ester	Iron chelator	TNBS-induced rat colitis	Jeong et al. (2015)
AKB-4924	Iron chelator	TNBS-induced mice colitis	Okumura et al. (2012)
CG-598	Unknown	DSS-induced mice colitis	Kim et al. (2021)
Betulinic acid hydroxamate	Dephosphorylation and inactivation of PHD2	TNBS- and DSS-induced mice colitis	Prados et al. (2021)

Gupta and co-workers (Gupta et al., 2014) demonstrated that, when orally administered, the PHD inhibitor TRC160334 had protective effects in TNBS and DSS mice colitis models. It was shown in Hep3B cells that TRC160334 had the ability to activate HIF-1α (Jamadarkhana et al., 2012). Jeong et al. (2015) reported that the iron chelator rosmarinic acid methyl ester inhibited PHD enzymatic activity and was able to ameliorate TNBS-induced colitis in rats, and the protection was associated with increased colonic HIF-1 activity. A recent study demonstrated that stabilization of HIF-1α by the PHD inhibitor CG-598 mitigated gut inflammation in DSS-induced colitis in mice (Kim et al., 2021).

AKB-4924 (also known as GB004), a predominant PHD inhibitor, was shown to protect against TNBS-induced mice colitis (Keely et al., 2014). This inhibitor did not have any protection of mice lacking HIF-1α in intestinal epithelial cells, indicating that epithelial HIF-1α is the target for AKB-4924-mediated protection. GB004 is an iron chelator and stabilizes HIF-1α by inhibiting PHD activity (Okumura et al., 2012). Oral administration of GB004 alleviated colonic inflammation with minor effects on protein levels of HIFα and expression of its target genes in extra intestinal organs, which limits the potential off-target effects (Marks et al., 2015). These results implicate that GB004 has preference for stabilization of HIF-1α within the gut and the intestinal epithelium is the central site of protection afforded by PHD inhibitor. HIF-1 stabilization by GB004 accelerated mice intestinal mucosal healing and reduced TNBS-induced colitis by inducing epithelial integrin expression (Goggins et al., 2021). GB004 exhibits protective effects directly on epithelial cells and drives protective effects on immune cells (Taylor et al., 2021). Administration of GB004 results in reduction in disease severity and improvements in histologic measures.

PHD inhibitors also play a role in alleviating intestinal fibrosis. It has been recently reported that PHD inhibition downregulates the expression of TGF-β1 in intestinal fibrosis (Manresa et al., 2016), indicating that the PHD inhibitors serve as anti-fibrotic agents in the treatment of IBD. Oral administration of betulinic acid hydroxamate (BAH), an inhibitor of PHDs, prevented TNBS- or DSS-induced mice colon inflammation and fibrosis (Prados et al., 2021). BAH-treated animals showed a significant reduction of fibrotic markers Tnc, Col1a2, Col3a1, Timp-1 and α-SMA and inflammatory markers F4/80+, CD3^+^, Il-1β and Ccl3 in colon tissue, as well as an improvement in epithelial barrier integrity and wound healing.

The aforementioned studies have demonstrated the therapeutic effects of PHD inhibitors in the treatment of animal gut inflammation. Notably, most of these studies showed the stabilization of HIF-1α and attributed the protection to the stabilization and activation of HIF-1. It should be noted that there is no conclusive demonstration that HIF-1α is the primary driver of the protective effects of these inhibitors. Inhibition of PHDs may also activate nuclear factor-kappa B (NF-κB) (Cummins et al., 2006; Welden et al., 2017). It was shown that ablation of NF-κB in epithelial cells resulted in serious chronic intestinal inflammation in mice (Nenci et al., 2007), indicating the requirement of NF-κB in the maintenance of the gut immune homeostasis. As NF-κB is a major regulator of immune and inflammatory processes (Nizet and Johnson, 2009; Capece et al., 2022), its activation induced by PHD inhibitors may also be of therapeutic benefit in IBD.

### 6.2 PHD inhibitor GB004 in clinical trail

As GB004 has been shown beneficial in treatment of intestinal inflammation in animal models, it is being investigated as a potential treatment option for IBD patients. A phase IA study showed that no serious adverse events were observed when the healthy subjects received a single ascending dose of GB004, indicating a well tolerance (Levesque et al., 2019) (Table 2).

**TABLE 2:** PHD inhibitor GB004 in clinical trail.

Clinical trail stage	Test population	Purpose	Results	References
Phase IA	Healthy people	Evaluation of the safety, tolerability, and pharmacokinetics of a single ascending dose of GB004	No serious adverse events	Levesque et al. (2019)
Phase IA	Healthy people	Evaluation of the safety, tolerability, and pharmacokinetics of multiple daily doses of GB004	Safe and tolerable	Levesque et al. (2020)
Phase IB	Patients with mild-to-moderate active UC	Evaluation of safety, pharmacokinetics, pharmacodynamics and efficacy of GB004	GB004 is safe, tolerable and beneficial in improving mucosal healing and reducing inflammation	Danese et al. (2022)

To determine the safety and pharmacokinetic profile, a multiple dose phase IA study was conducted in healthy people in Canada (Levesque et al., 2020). It is randomized, double-blinded and placebo-controlled. GB004 solution or placebo solution were orally administered at three doses once a day for 8 days. Forty-two people participated and there were no recorded serious adverse events. Following oral administration, the drug was absorbed and eliminated quickly from the systemic circulation. The influences of GB004 and placebo on levels of erythropoietin (EPO) and VEGF in plasma were similar with no dose-related effects, which highlights the predominant effect of GB004 on the intestine. The data indicated that GB004 at the doses tested did not influence the expression of EPO and VEGF in plasma. These results might be due to low accumulation of GB004 in plasma. The biopsie assay indicated that there was more GB004 in colon than in the plasma (Levesque et al., 2020). These results of the study suggest that GB004 at the doses administrated daily are safe and tolerable.

A first-in-patient, phase IB, double-blinded, placebo-controlled study was performed to evaluate the safety, tolerability and pharmacokinetics of GB004 (Danese et al., 2022). Thirty-four adult participants that had mild-to-moderate active UC were randomized to GB004 solution (120 mg) (n = 23) vs. placebo (n = 11) once daily for 28 days. After GB004 treatment, a greater proportion of the patients had reduced faecal calprotectin and mucosal healing. Formation of faecal calprotectin is induced in inflammation and its expression level correlates with disease activity and it is used as a clinical biomarker for mucosal inflammation (Jukic et al., 2021). GB004 was generally well tolerated when administered orally at 120 mg once daily for 28 days. There were no discernable difference between the treatment groups regarding systemic levels of EPO and VEGF. The concentrations of GB004 in colonic tissues on day 28 were greater than those in plasma (approximately 6 and 65 times higher than peak and average plasma concentrations). The substantially higher contents of GB004 in colon relative to those in plasma indicates a local gut effect of GB004, which may explain the absence of increased systemic levels of EPO and VEGF relative to placebo.

Hypoxia promotes glycolysis and thus excess lactate production, leading to acidification of the extracellular microenvironment, which may influence the therapeutic efficacy of drugs (Singh et al., 2021). This phase IB trial study showed the therapeutic benefits of GB004 in the treatment of UC, suggesting that this compound works in lactate acidosis.

Currently, a larger phase 2 SHIFT-UC study (NCT03860896) about GB004 on active UC is ongoing.

### 6.3 PHD inhibitors development and limitation

Several PHD inhibitors have been developed and their potential in treating IBD and other diseases such as anemia associated with chronic kidney disease (CKD) are under investigation (Welden et al., 2017; Semenza, 2019). Enhanced angiogenesis and increased expression of EPO were observed in conditional knockout of PHD2 (Takeda et al., 2006; Takeda et al., 2007; Katschinski, 2009). These findings and previous results that HIF-induced EPO production and concomitantly enhanced erythropoiesis (Semenza and Wang, 1992) imply that HIF activation by inhibiting PHDs is favorable to people with anemia and ischemia-related diseases. Pharmacological manipulation of PHD-HIF axis has been quested for treating disorders related to local and systemic hypoxia. PHD inhibitors were developed for the treatment of chronic kidney disease (CKD)-related anemia (Welden et al., 2017; Semenza, 2019; Wish et al., 2021; Macdougall, 2022). At least six PHD inhibitors roxadustat (FG-4592), daprodustat (GSK1278863), vadadustat (AKB-6548), molidustat (BAY 85-3934), enarodustat (JTZ-951) and desidustat have been developed and the phase 3 clinical trials showed that their effects were non-inferior to current EPO-stimulating agents (Sugahara et al., 2022). Roxadustat was first approved in China for treatment of CKD-related anemia patients receiving hemodialysis or peritoneal dialysis in 2018 (Dhillon, 2019) and for the treatment of CKD-related anemia patients not receiving dialysis in 2019 (Li et al., 2020). Roxadustat was then launched in other countries such as Japan, Chile, South Korea, the European Union, and the United Kingdom (Sugahara et al., 2022). On 01 Feb 2023, FDA approved daprodustat as the first oral treatment for anemia caused by CKD for adults who have been receiving dialysis for at least 4 months (https://www.fda.gov/).

Though no adverse effects were observed in studies of GB004 in IBD patients, there are not any long-term clinical data with GB004. The worry about the long-term use of PHD inhibitors are raised (Welden et al., 2017). It is well known that HIFα is highly expressed in cancer cells and its expression is positively linked with cancer aggressiveness and mortality (Schito and Semenza, 2016). Thus, the risk of HIF-activating therapies to promote tumor should be assessed. Activation of HIFα promotes EPO expression and subsequent erythrocyte formation. The agents that stimulate EPO expression are associated with an increased risk of thromboembolic diseases (Vittori et al., 2021; Semenza, 2022). The risk of fibrosis is another concern when using inhibitors of PHD. Intestinal fibrosis is a common complication of IBD (D'Alessio et al., 2022). More than 30% of IBD patients have intestinal fibrosis (Ramakrishnan and Shah, 2016). It was demonstrated that activation of HIF1α in epithelial cells promoted fibrogenesis *in vivo*. Higgins et al. (2007) provided clinical and genetic evidence that HIF-1 activation in renal epithelial cells might promote fibrogenesis through the induction of extracellular matrix-modifying factors and lysyl oxidase. The disturbance of biochemical processes by inhibition of PHDs is also a concern. Currently, some of the tested PHD inhibitors are analogues of 2-OG. In humans, there are many 2-OG-dependent dioxygenases and these enzymes hydroxylate proteins involved in many biological processes such as collagen and hormone synthesis and fatty acid metabolism (McDonough et al., 2010; Markolovic et al., 2015; Losman et al., 2020). The use of 2-OG analogues might potentially influence these reactions and result in side effects. Some PHD inhibitors are Fe^2+^ chelators and may inhibit other enzymes requiring Fe^2+^, which could also lead to unwanted adverse effects.

## 7 Conclusion

IBD is a chronic inflammatory disorder of intestine and is characterized by disrupted intestinal barrier and dysregulated immune. HIFα plays a critical role in protecting intestinal epithelial barrier and maintaining the healthy mucosal function. Many studies have demonstrated that stabilization of HIFα through inhibition of PHDs is protective in experimental colitis. Targeting PHD-HIF system to repair the disrupted intestinal barrier is becoming a novel strategy for therapy of IBD. A few PHD inhibitors have proven to be beneficial in several models of IBD and clinical trials are ongoing. As there is potential side effect for persistent activation of HIFs, systemic exposure to PHD inhibitors may cause adverse effect. Thus, a long-term follow-up is required to confirm the safety of the treatment with PHD inhibitors. The potential side effects of PHD inhibitors should be assessed before clinical use. Developing PHD inhibitors that are intestine preferential localization might be an approach to reduce the adverse effect. In summary, development of the inhibitors targeting PHDs may meet the unmet needs for IBD treatment and will have deep impact on medicine.
